# The Proximity of PD-1^−^CD103^+^ Tissue-Resident CD8^+^ T Cells to Tumor Cells Is Correlated with Improved Clinical Outcomes in Patients with Cholangiocarcinoma

**DOI:** 10.3390/cancers18040680

**Published:** 2026-02-19

**Authors:** Zhenyu Li, Danping Liu, Jingjing He, Junrui Ma, Muyuan He, Xiaobao Yang, Yanan Zhao, Xuefeng Fei, Dakang Xu, Mengjie Deng

**Affiliations:** 1 Department of Laboratory Medicine, Ruijin Hospital, Shanghai Jiao Tong University School of Medicine, Shanghai 200025, China; 2 College of Health Sciences and Technology, Shanghai Jiao Tong University School of Medicine, Shanghai 200025, China

**Keywords:** CD103^+^CD8^+^ tissue-resident T cells, spatial interaction, G-cross, PD-1 expression, cholangiocarcinoma

## Abstract

**Background/Objectives:** Cholangiocarcinoma (CCA) is characterized by a heterogeneous immune microenvironment, where the prognostic significance of CD8^+^ tissue-resident memory T (T_RM_) cell activation and spatial positioning remains to be fully elucidated. This study investigated how the activation phenotypes and their spatial distribution relative to tumor cells influence anti-tumor immunosurveillance and predict clinical outcomes in CCA. **Methods:** Multiplex immunohistochemistry (mIHC) and single-cell RNA sequencing (scRNA-seq) were employed to characterize naïve (PD-1^−^CD103^+^CD8^+^) and exhausted (PD-1^+^CD103^+^CD8^+^) subsets. G-cross function analysis was utilized to quantify the spatial proximity between these specific T_RM_ subsets and tumor cells, correlating the spatial interaction with patient overall survival. **Results:** scRNA-seq profiling revealed that PD-1^−^CD103^+^CD8^+^ T_RM_ cells were enriched in genes associated with lymphocyte activation and cytotoxicity, while PD-1^+^CD103^+^CD8^+^ T_RM_ cells exhibited an exhaustion signature. Spatially, PD-1^−^CD103^+^CD8^+^ T_RM_ cells exhibited increased interactions with tumor cells, whereas PD-1^+^CD103^+^CD8^+^ T_RM_ cells showed reduced engagement. Therefore, the close proximity of PD-1^−^CD103^+^CD8^+^ T_RM_ cells to tumor cells was identified as a significant predictor of favorable clinical outcomes. **Conclusions:** The activation state of CD8^+^ T_RM_ cells combined with their spatial localization constitutes a critical prognostic factor in CCA. Effective anti-tumor immunosurveillance relies on the direct engagement of naïve T_RM_ cells with tumor cells. These findings highlight the potential of PD-1-targeted immunotherapies to remodel the spatial proximity of the tumor microenvironment, potentially promoting the redistribution of effector cells into tumor-proximal regions.

## Simple Summary

Cholangiocarcinoma presents a clinical challenge due to its immunosuppressive tumor microenvironment and therapeutic resistance. This study investigated how the spatial positioning of tissue-resident memory T cells relative to tumor cells is related to anti-tumor immunity and patient survival. We aimed to determine whether the proximity of these immune cells to malignant cells is correlated with patient survival. These findings indicate that the spatial organization of CD8^+^ tissue-resident memory T cells is a critical prognostic factor, suggesting that effective immune surveillance requires not only the presence of specific T cell subsets but also their precise localization against tumor cells. These insights support the development of immunotherapies that promote effector cell infiltration and engagement within the cholangiocarcinoma tumor microenvironment.

## 1. Introduction

Cholangiocarcinoma (CCA) is a malignancy characterized by a highly heterogeneous and immunosuppressive tumor microenvironment (TME) [1,2]. Within this complex ecosystem, CD8^+^ T lymphocytes are associated with anti-tumor immune responses [3]. However, their functional efficacy is not uniform and appears to be influenced by their differentiation state, activation status, and spatial localization within tumor tissues [4,5]. CD8^+^ T cells expressing programmed cell death protein 1 (PD-1) are primarily memory subsets that have undergone antigen exposure [4]. According to their migratory characteristics, they can be classified into CD8^+^ effector memory T (T_EM_) cells and CD8^+^ tissue-resident memory T (T_RM_) cells. T_EM_ cells circulate between the blood and peripheral tissues [5]. T_RM_ cells are defined by their permanent residency in tissues and their independence from circulatory T cell populations [6,7]. Additionally, tumor-associated T_RM_ lymphocytes express surface markers of lymphocyte exhaustion, such as PD-1, TIM3, and CD39 [8]. Although T_RM_ lymphocytes express the PD-1 molecule, they differ from those in a state of terminal exhaustion. After reactivation, they have been reported to retain their proliferative capacity and cytotoxic potential, which may contribute to antigen-specific immune responses [9,10,11].

CD8^+^ T_RM_ cells are defined by specific migratory properties and express adhesion molecules (such as CD69 and CD103) [12]. Although tissue residence is regulated by a conserved transcriptional program and mediates the expression of necessary adhesion molecules, such as CD103, there is still heterogeneity in CD8^+^ T_RM_ cells in different organs [13]. Specific molecules are required to facilitate the residence of T_RM_ cells in various organs. For example, the molecular characteristics of cholangiocarcinoma are not yet fully clarified [14]. CD8^+^ T_RM_ cells are targeted by surface CD103 and bind to the E-cadherin on the surface of tumor cells, which may facilitate their retention within the TME and support prolonged tissue residence. Thus, such proximity could provide a spatial context conducive to rapid effector functions following reactivation [15,16].

A critical, yet understudied, aspect of T_RM_ biology in cancer is the interplay between their functional states, defined by markers such as PD-1, and their precise spatial relationship with tumor cells [17]. While the mere presence of T_RM_ cells may be beneficial, their anti-tumor activity may depend on close spatial proximity to exert cytotoxic effects [18]. Therefore, we hypothesized that not all CD103^+^CD8^+^ T_RM_ cells are functionally equivalent in CCA and that their associations with clinical outcomes may vary according to a combination of their activation or exhaustion phenotypes (e.g., PD-1 expression) and their spatial distributions relative to those of malignant cells.

To test this hypothesis, we employed an integrative approach combining high-plex multiplex immunohistochemistry (mIHC) for spatial phenotyping and single-cell RNA sequencing (scRNA-seq) for deep transcriptional profiling. This study aims to: (1) characterize the heterogeneity of CD8^+^ T cells, particularly CD103^+^ T_RM_ subsets, within the CCA TME; (2) classify T_RM_ cells based on PD-1 co-expression; (3) quantify the spatial interactions between distinct T_RM_ subsets and tumor cells; and (4) evaluate the associations of these spatial-functional phenotypes with clinical outcomes. Our findings offer insights into the spatial dynamics of immunosurveillance in CCA and may inform strategies for predicting or improving responses to PD-1 blockade.

## 2. Materials and Methods

### 2.1. Patient Cohorts and Data Acquisition

This study employed two independent cohorts: a public scRNA-seq cohort and an institutional tissue microarray (TMA) cohort. The scRNA-seq data were obtained from the Genome Sequence Archive (GSA) database at the National Genomics Data Center (HRA000863) to analyze cells isolated from intrahepatic cholangiocarcinoma (iCCA), including tumor cells, CD45^+^ immune cells, and other stromal cells.

The human iCCA specimens were obtained from Ruijin Hospital, Shanghai Jiao Tong University School of Medicine (Shanghai, China). Formalin-fixed paraffin-embedded (FFPE) tissue blocks were sectioned for hematoxylin and eosin (H&E) staining. Pathology was independently verified by two qualified pathologists. Representative tumor regions and matched adjacent-normal tissues identified by H&E were selected for TMA construction. The TMA comprised 140 cores derived from 70 tumor–normal pairs. Among these, 67 patients with complete clinical information were pathologically confirmed iCCA and had undergone curative resection; after excluding samples with poor tissue quality, 61 pairs were ultimately analyzed. The detailed clinicopathological characteristics are summarized in Table S1. (Female/Male: 29/38; Age < 65/≥65 years: 42/25; TNM stages [AJCC 8th edition]: Stage I, n = 16; Stage II, n = 23; Stage III, n = 13; Stage IV, n = 15). Written informed consent was obtained from all the subjects prior to enrollment. This study was conducted in strict accordance with the Declaration of Helsinki and was approved by the Institutional Ethics Committee of Ruijin Hospital, Shanghai Jiao Tong University School of Medicine (Approval No. 2021-207).

### 2.2. Single-Cell RNA-Seq Data Processing and Annotation

Raw scRNA-seq data from 14 patients (paired tumor and adjacent normal tissues) were processed via 10X Cell Ranger (v9.0.1), which involved aligning them to the human reference genome (GRCh38-2024A, provided by 10X Genomics), and creating a gene-cell unique molecular identifier (UMI) matrix. All 28 samples were merged into a dataset of 343,170 cells. Quality control, normalization, and downstream analysis were processed via Seurat v5 (v5.2.1, R package). Stringent quality control was performed based on the number of RNA features between 200 and 3000, the percentage of mitochondrial genes < 10%, and the proportion of erythroid genes less than 1%. The cells that passed the QC were normalized using the recommended settings and the “vst” method, and 3000 highly variable genes were identified. Cell cycle score was calculated using the “CellCyclesScoring” algorithm and normalized by “ScaleData” function. Dimensionality reduction was conducted using Principal Component Analysis (PCA) followed by Uniform Manifold Approximation and Projection (UMAP) for cell clustering. Major cell types were annotated using canonical lineage markers, including T cells (CD3E, CD4, CD8A), B cells (CD19, MS4A1, CD79A), NK cells (FCGR3A, NCAM1), and myeloid cells (CD14, CD68, CD1C, CSF3R). Specifically, CD8^+^ T cells were subclustered to distinguish between functional phenotypes, including naïve (CCR7^+^, SELL^+^), effector (GZMB^+^, PRF1^+^), and tissue-resident memory (CD103^+^, ZNF683^+^) subsets. Further trajectory and subtype analyses focused on distinguishing naïve T_RM_ (PD-1^−^CD103^+^) from exhausted T_RM_ (PD-1^+^CD103^+^) subsets to evaluate their distinct functional divergence.

### 2.3. Transcriptional Signature and Functional Enrichment Analysis

Differential expression analysis was performed using the “FindAllMarkers” function. DEGs were identified between the PD-1^+^ and PD-1^−^ T_RM_ clusters to characterize their activation and exhaustion profiles. GO enrichment analysis was conducted to annotate the DEGs, focusing on Biological Processes (BP), Cellular Components (CC), Molecular Functions (MF) and immune response genes [19,20]. Pathways related to immune cell activation, leukocyte-mediated immunity, and immune effector processes were specifically analyzed.

### 2.4. Gene Set Enrichment Analysis

To calculate the signature scores, the average expression of the signature genes was calculated based on the normalized matrix generated by the Seurat package. GSEABase (v1.68.0, R package) was employed to conduct functional enrichment analysis for the defined gene signatures and the cluster-specific DEGs identified from previous analyses (PD-1^−^CD103^+^CD8^+^ T cells vs. PD-1^+^CD103^+^CD8^+^ T cells). The cytotoxicity and exhaustion scores were determined by calculating the average expression of the published signature genes [21]. Gene sets with an adjusted *p*-value < 0.05 were selected as functionally enriched biological states or processes. The positive normalized enrichment score (NES) indicates that a particular functional gene set is enriched at the beginning of the sorting sequence, suggesting upregulation in the specific cell population compared with other cells. Conversely, a negative NES implies that a specific functional gene set is enriched at the end of the sorting sequence, suggesting downregulation in the targeted cell population compared with other cells.

### 2.5. Multiplex Immunohistochemistry (mIHC)

Multiplex immunofluorescence staining was performed on 4-µm thick FFPE TMA sections to visualize the spatial arrangement of tumor and immune cells. The slides were heated at 60 °C for 1 h to enhance tissue adhesion, followed by deparaffinization in xylene and rehydration through a graded ethanol series (100%, 95%, 90%, 85%, 50%, ddH_2_O). Heat-induced epitope retrieval was optimized for each antibody using Tris-EDTA (pH 9.0) for anti-CD8a, anti-CD69, anti-CD103, and anti-pan-keratin antibodies, and citrate buffer (pH 6.0) for anti-PD-1 and anti-TCF-1 antibodies. Endogenous peroxidase activity was blocked, and nonspecific binding was reduced with a protein blocking buffer. Sections were then incubated with the primary antibody panel (see Table S2). Signal detection was achieved via Opal-tyramide signal amplification (TSA) with distinct fluorophores (Opal 480, 520, 570, 620, 690, 780). Cell nuclei were counterstained with DAPI, and slides were mounted for imaging. Whole-slide multispectral images were acquired via the Vectra Polaris Quantitative Pathology Imaging System, and spectral unmixing was performed with inForm software (v2.6.0) to isolate specific signals for analysis.

### 2.6. Spatial Analysis

Spatial analysis was performed via APTIME software (v3.0.0.3666) to achieve precise tissue segmentation and cellular phenotyping based on nuclear DAPI staining. A multiplex marker panel comprising PanCK, CD8, CD103, PD-1, CD69, and TCF-1 was employed to distinguish tumor and immune cell subsets, with positivity thresholds calibrated against negative controls and validated by pathological review. Phenotypes were defined via hierarchical gating of the mean fluorescence intensity to identify tumor cells (PanCK^+^), cytotoxic T cells (CD8^+^), tissue-resident T cells (CD8^+^CD103^+^), and exhausted T_RM_ cells (PD-1^+^CD103^+^CD8^+^). Following automated cell detection and spatial coordinate extraction, tissue regions were segmented into intra-tumor compartments defined by PanCK^+^ tumor regions and stromal compartments comprising the surrounding PanCK^−^ areas. To quantify the spatial distribution of immune cells, the proportion of target cells within a 100-µm zone from the tumor boundary was calculated by dividing the region into sequential 10-µm distance bands, enabling a graded assessment of infiltration depth. Direct cellular interactions were evaluated by counting target immune cells located within a 10 µm radius of each reference tumor cell. Additionally, the G-cross function was applied to model the probability of immune cell occurrence within a specified radius of tumor cells, providing a robust statistical characterization of spatial infiltration patterns.

### 2.7. Statistical Analysis

All the statistical analyses were performed via R software (v4.4.2), Python (3.10) and GraphPad Prism (v10.4.1). Group comparisons for continuous variables were performed using the Wilcoxon rank-sum test for unpaired data or paired t-tests for matched samples, as appropriate. OS was estimated by the Kaplan-Meier method, and group differences were assessed with the log-rank test. Univariate and multivariate Cox proportional hazards models were employed to identify independent prognostic factors, with results expressed as HR and 95% confidence intervals (CI). The variables included in the models were age, sex, TNM stage, and densities of immune cell subsets. A two-sided *p*-value less than 0.05 was considered statistically significant.

## 3. Results

### 3.1. Spatial Landscape and Heterogeneity of CD8^+^ T Cell Infiltration in CCA

**Figure 1:**
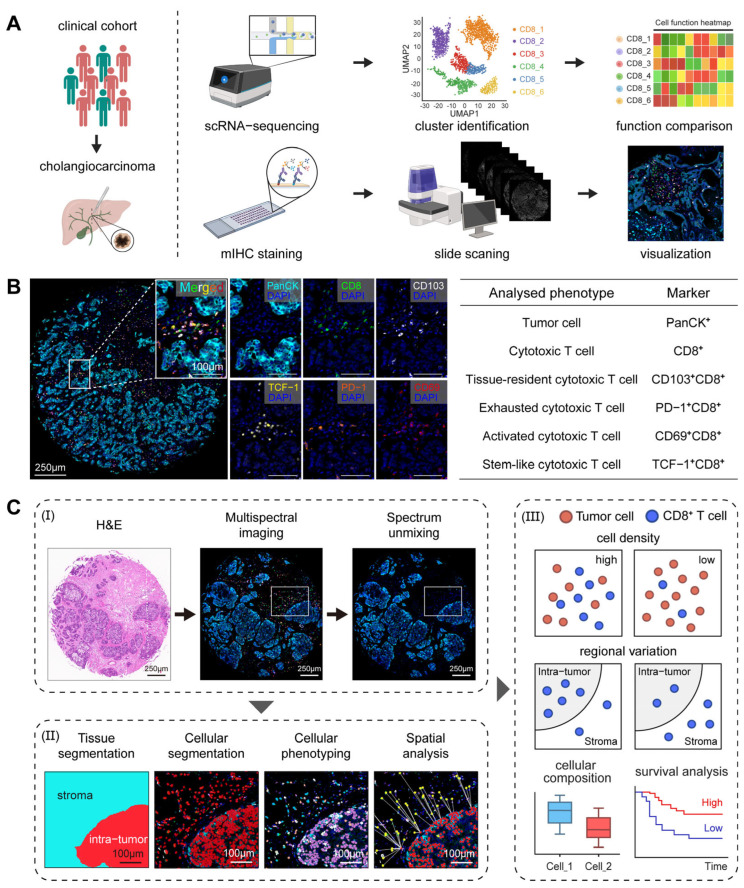
Multiplex immunohistochemistry (mIHC) workflow and spatial analysis pipeline in CCA. (**A**) Schematic overview of the study design. The integrated analysis combined the processing of a scRNA-seq dataset with mIHC staining performed on a clinical cohort of CCA patients. (**B**) Representative mIHC staining image and table defining the analyzed cell phenotypes based on marker expression. (**C**) Image analysis pipeline comprising multispectral imaging, spectrum unmixing, and automated segmentation of tissue and single cells. Downstream analysis integrates cellular phenotyping with spatial quantification and survival correlation.

Recent progress has revealed the molecular diversity of CCA. Nevertheless, the spatial architecture and cellular crosstalk within the TME remain poorly understood. To characterize the cellular composition, spatial distribution, and molecular variation systematically, we developed an integrated analytical pipeline combining mIHC with scRNA-seq to investigate the spatial heterogeneity of the immune landscape in CCA (Figure 1A). About these two independent cohorts, mIHC was performed on 67 primary iCCA samples; scRNA-seq was derived from 14 iCCA patients with paired tumor and adjacent normal tissues. A comprehensive antibody panel (PanCK, CD8, CD103, PD-1, CD69, TCF-1) was employed to concurrently profile tumor cells and major functional subsets of CD8^+^ T cells, offering a representative characterization of critical cellular interactions and spatial architecture within the TME (Figure 1B). The image analysis pipeline facilitated precise spectrum unmixing, tissue segmentation, and cellular phenotyping (Figure 1C). Key subsets were identified and categorized as tumor cells (PanCK^+^), cytotoxic (CD8^+^), tissue-resident cytotoxic (CD103^+^CD8^+^), exhausted cytotoxic (PD-1^+^CD8^+^), activated cytotoxic (CD69^+^CD8^+^), and stem-like cytotoxic (TCF-1^+^CD8^+^) T cells (Figure 1B). This approach allowed for the systematic evaluation of the heterogeneous infiltration patterns of these subsets across intra-tumor and stroma regions.

### 3.2. Spatial Profiling of CD8^+^ T Cells Revealed Distinct Prognostic Value of CD8^+^ T Cells Subsets

**Figure 2:**
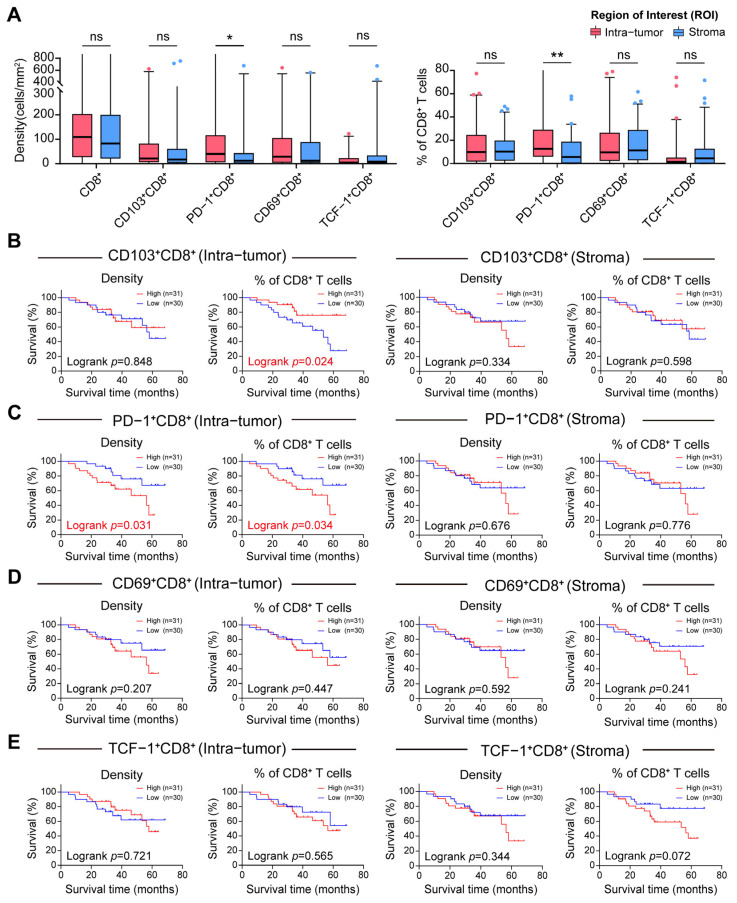
Intra-tumor infiltration of CD103^+^ tissue-resident and PD-1^+^ exhausted CD8^+^ T cells exerts differential clinical outcomes. (**A**) Box plots comparing the density (cells/mm^2^) and percentage (/CD8^+^) of total CD8^+^, CD103^+^CD8^+^, PD-1^+^CD8^+^, CD69^+^CD8^+^, and TCF-1^+^CD8^+^ T cells between intra-tumor (red) and stroma (blue) compartments defined by Region of Interest (ROI) image segmentation. Statistical significance was determined by paired t-test (* *p* < 0.05, ** *p* < 0.01, ns: not significant). Dots in the figure represent outliers. (**B**–**E**) Kaplan-Meier survival curves stratified by the density (**left**) and percentage (**right**) of specific T cell subsets within the intra-tumor (**left panel**) and stroma (**right panel**) regions. (**B**) High intra-tumor percentage of CD103^+^CD8^+^ T cells was associated with favorable survival (*p* = 0.024). (**C**) Both high density and percentage of intra-tumor infiltration of PD-1^+^CD8^+^ T cells significantly correlated with poor prognosis (*p* < 0.05). (**D**,**E**) CD69^+^CD8^+^ and TCF-1^+^CD8^+^ subsets showed no significant prognostic associations in either compartment.

Based on the TMA cohort, the prognostic significance of distinct T cell phenotypes was investigated based on their location in the intra-tumor and stroma regions in CCA. CD8^+^ T cells in tissue samples were classified according to the absolute cell density measured in cells per square millimeter and the proportion of each T cell subset relative to the total CD8^+^ T cell population in each compartment (Figure 2A; Figure S1). For tissue-resident cytotoxic T cells (CD103^+^CD8^+^), the clinical outcomes were primarily associated with the relative abundance of these cells within the intra-tumor region, rather than their absolute density or presence in the stroma. Neither the intra-tumor nor the stroma density was significantly associated with survival. Similarly, the proportion of CD103^+^CD8^+^ cells in the stroma did not exhibit any prognostic significance. However, a greater proportion of CD103^+^CD8^+^ cells in the intra-tumor compartment was associated with a favorable prognosis, suggesting a potential association between intra-tumoral T_RM_ cells and improved outcomes (Figure 2A,B; Figure S2). In contrast, exhausted cytotoxic T cells (PD-1^+^CD8^+^) were associated with reduced survival, particularly within the intra-tumor compartment. Specifically, a greater density of PD-1^+^CD8^+^ cells in tumor islands, as well as a greater proportion of exhausted cells among total intra-tumor CD8^+^ T cells, correlated with reduced overall survival. Notably, this adverse prognostic impact was spatially limited to the intra-tumor region, as neither the density nor the percentage of PD-1^+^CD8^+^ cells in the stroma was correlated with patient survival (Figure 2A,C; Figure S2). Other functional T cell subsets, including activated (CD69^+^CD8^+^) and stem-like (TCF-1^+^CD8^+^) cytotoxic T cells, did not show any statistically significant prognostic value in either region (Figure 2D,E; Figure S2). Collectively, these findings suggest that CCA patient survival is associated with the balance between the percentages of functional tissue-resident (CD103^+^) and exhausted (PD-1^+^) T cells within the intra-tumor region.

### 3.3. Classification of CD103^+^CD8^+^ T_RM_ Cells into Naïve and Exhausted Subsets

**Figure 3:**
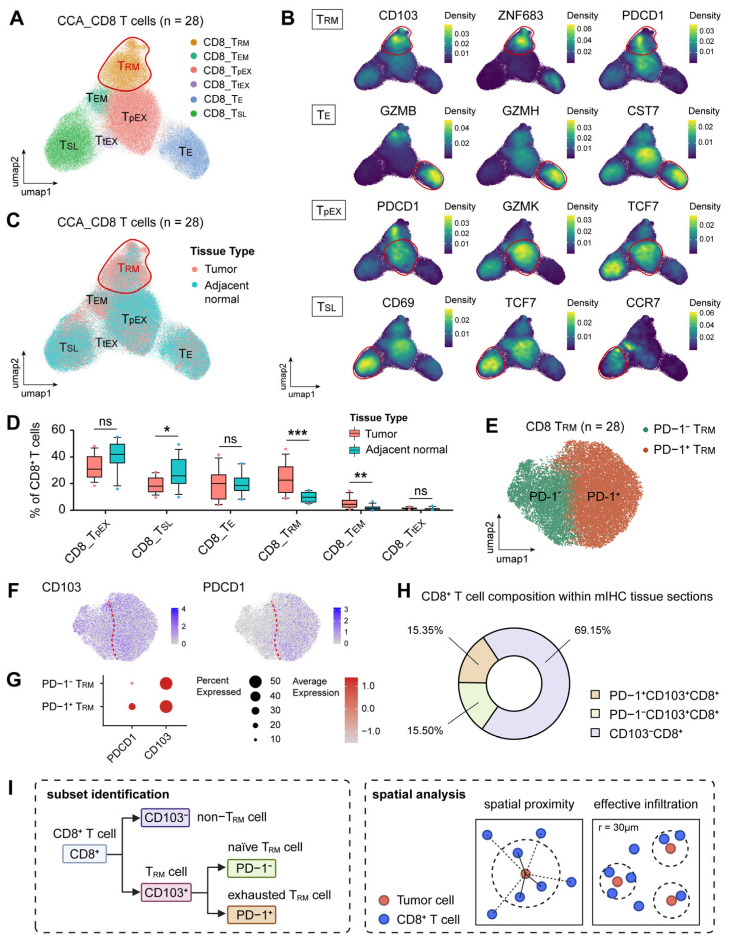
Single-cell RNA sequencing (scRNA-seq) identifies and characterizes the heterogeneity of T_RM_ cells in CCA. (**A**) UMAP visualization of 119,381 CD8^+^ T cells from 28 CCA samples colored according to annotated subsets including CD8 tissue-resident memory (CD8_T_RM_), CD8 effector memory (CD8_T_EM_), CD8 progenitor exhausted (CD8_T_pEX_), CD8 terminally exhausted (CD8_T_tEX_), CD8 effector (CD8_T_E_), and CD8 stem-like (CD8_T_SL_) T cell populations. (**B**) UMAP feature plots displaying the expression density of key marker genes across all subsets. (**C**) UMAP plot split by tissue origin showing the distribution of cells in tumor versus adjacent normal tissues. (**D**) Box plots quantifying the percentage of each CD8^+^ T cell subset in tumor versus adjacent normal tissues where CD8_T_RM_ and CD8_T_EM_ were significantly enriched in the tumor while CD8_T_SL_ cells were decreased. Statistical significance was determined by paired t-test (* *p* < 0.05, ** *p* < 0.01, *** *p* < 0.001, ns: not significant). (**E**) Subclustering analysis of the CD8_T_RM_ population resolving two transcriptionally distinct subsets annotated as PD-1^−^ and PD-1^+^ T_RM_ subsets. (**F**) UMAP feature plots delineating the differential expression distributions of CD103 and PDCD1 markers within the T_RM_ subsets. (**G**) Dot plot quantifying the expression frequency and average intensity of PDCD1 and CD103 across the identified T_RM_ subsets. (**H**) Pie chart illustrating the overall composition of the CD8^+^ T cell population. The population was categorized into non-T_RM_ cells (CD103^−^CD8^+^, 69.15%), naïve T_RM_ cells (PD-1^−^CD103^+^CD8^+^, 15.50%), and exhausted T_RM_ cells (PD-1^+^CD103^+^CD8^+^, 15.35%). (**I**) Schematic diagram establishing the stratification of CD103^+^CD8^+^ T_RM_ cells into naïve T_RM_ and exhausted T_RM_ phenotypes for downstream spatial interrogation.

To further investigate the functional heterogeneity within the tissue-resident memory (T_RM_) compartment, unsupervised clustering analysis was conducted on CD8^+^ T cells from the scRNA-seq cohort, encompassing 28 samples (tumor and adjacent normal tissues) derived from 14 iCCA patients (Figure 3A).

Multiple CD8^+^ T cell subsets were identified and labeled according to the expression of canonical marker genes, including CD8^+^ tissue-resident memory (CD8_T_RM_), CD8^+^ effector memory (CD8_T_EM_), CD8^+^ progenitor exhausted (CD8_T_pEX_), CD8^+^ terminally exhausted (CD8_T_tEX_), CD8^+^ effector (CD8_T_E_), and CD8^+^ stem-like (CD8_T_SL_) T cell populations (Figure 3A,B). The distribution of these CD8^+^ T cell subsets was quantified and compared between tumor and adjacent normal tissues (Figure 3C). A quantitative analysis of T cell subsets percentages demonstrated significant differences between the tumor and adjacent normal microenvironments (Figure 3D). The CD8_T_RM_ subset was notably enriched in tumor tissues compared with adjacent normal tissues (*p* < 0.001). Similarly, CD8_T_EM_ cells were present at a significantly greater frequency within the tumor compartment (*p* < 0.01). In contrast, the CD8_T_SL_ subset was significantly reduced in tumor tissues (*p* < 0.05). No statistically significant differences were detected in the CD8_T_pEX_, CD8_T_E_, or CD8_T_tEX_ subsets between these two tissue types (Figure 3D). Subclustering of the CD8_T_RM_ subset further identified two distinct functional subclusters (Figure 3E). Both functional subclusters highly expressed CD103. Nevertheless, one subcluster presented high PD-1 expression, whereas the other presented low PD-1 expression in comparison (Figure 3F,G). When examining the mIHC tissue microarray, it can also be noted that CD8^+^ T cells in tumor tissues are composed of two T_RM_ subsets (PD-1^−^ and PD-1^+^) and a CD103^−^ non-T_RM_ subset (Figure 3H). Based on these findings, we suggest a workflow to further categorize T_RM_ subsets into PD-1^−^ naïve T_RM_ and PD-1^+^ exhausted T_RM_ subsets (based on CD103^+^ expression) and conduct spatial analysis from the aspects of spatial proximity and effective infiltration (Figure 3I).

### 3.4. Discrepant Functional States and Spatial Characteristics of T_RM_ Subsets

**Figure 4:**
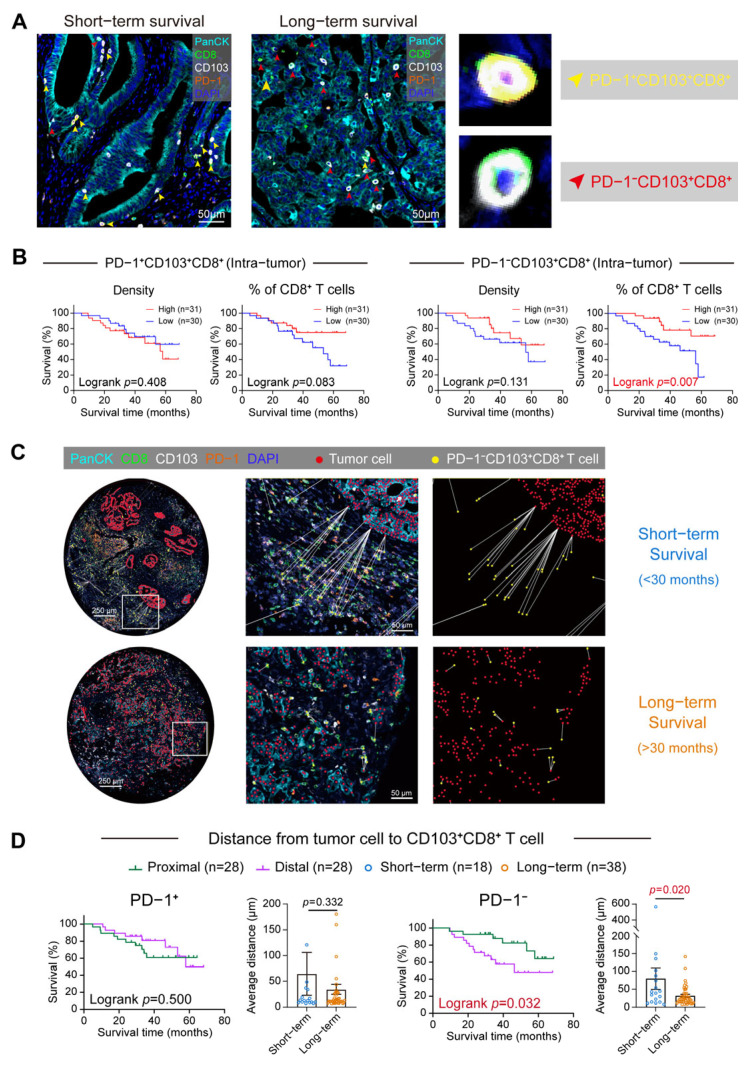
PD-1 expression delineates T_RM_ subsets with distinct functional states, spatial distributions, and clinical impacts. (**A**) Representative mIHC images distinguishing the spatial distribution of PD-1^+^ and PD-1^−^ T_RM_ cells in long-term versus short-term survivors, where yellow and red arrowheads indicate PD-1^+^ and PD-1^−^ T_RM_ subsets, respectively. (**B**) Kaplan-Meier survival analysis stratified by intra-tumor density and percentage relative to the total CD103^+^CD8^+^ population demonstrated that a high percentage of PD-1^−^ T_RM_ cells conferred a significant survival advantage, whereas a high percentage of PD-1^+^ T_RM_ cells was associated with a poor prognosis. (**C**) Visualization of the spatial distance analysis depicting distance measurements from tumor cells to T cells alongside representative images for distinct survival groups. For spatial analysis algorithm requirements, quality control excluded samples with fewer than 1500 combined tumor cells and CD8^+^ T cells within tumor tissues (n = 5). (**D**) Kaplan-Meier survival curves based on spatial proximity indicate that the close proximity of PD-1^−^ T_RM_ cells to tumor cells significantly predicts prolonged survival, whereas the spatial distribution of PD-1^+^ T_RM_ cells shows no prognostic relevance; the bar graph depicts the average distance of naïve and exhausted T_RM_ subsets to tumor cells in distinct survival groups.

The clinical relevance of the transcriptomically defined T_RM_ subsets was validated using mIHC to map the in situ distribution of exhausted (PD-1^+^CD103^+^CD8^+^) and naïve (PD-1^−^CD103^+^CD8^+^) T_RM_ populations (Figure 4A; Figure S3). Survival analysis demonstrated that the percentage of the exhausted T_RM_ subset among CD8^+^ T cells showed no statistically significant association with overall survival (Logrank *p* = 0.083). In contrast, a higher percentage of the naïve T_RM_ subset was significantly correlated with favorable outcomes (Logrank *p* = 0.007, Figure 4B), demonstrating stronger statistical significance than the unstratified T_RM_ population (Logrank *p* = 0.024, Figure 2B). Notably, the absolute densities of both subsets lacked prognostic significance. Distinct spatial patterns were confirmed by representative immunofluorescence images (Figure 4A). A predominance of naïve T_RM_ cells was shown in long-term patients and an accumulation of exhausted T_RM_ cells was observed in short-term patients. The spatial immune-tumor interactions were further quantified by measuring the physical distance between tumor cells and T_RM_ subsets (Figure 4C). It was observed that close spatial proximity of naïve T_RM_ cells to tumor cells was associated with a significantly prolonged survival (Logrank *p* = 0.032, Figure 4D). However, the proximity of exhausted T_RM_ cells to tumor cells showed no significant correlation with survival (Logrank *p* = 0.500, Figure 4D). Our results may suggest that a subset of T_RM_ cells stably reside in non-lymphoid peripheral tissues and may contribute to local immune responses independently of circulating T cells. The abundance and the proximity of naïve T_RM_ (PD-1^−^) cells to tumor cells may reflect their potential involvement in anti-tumor immune responses. Additionally, PD-1^+^ T_RM_ cells, which express surface markers associated with T cells exhaustion, exhibit molecular features consistent with functional impairment.

### 3.5. Spatial Proximity of PD-1^−^ T_RM_ to Tumor Cells Predicts Favorable Outcomes

**Figure 5:**
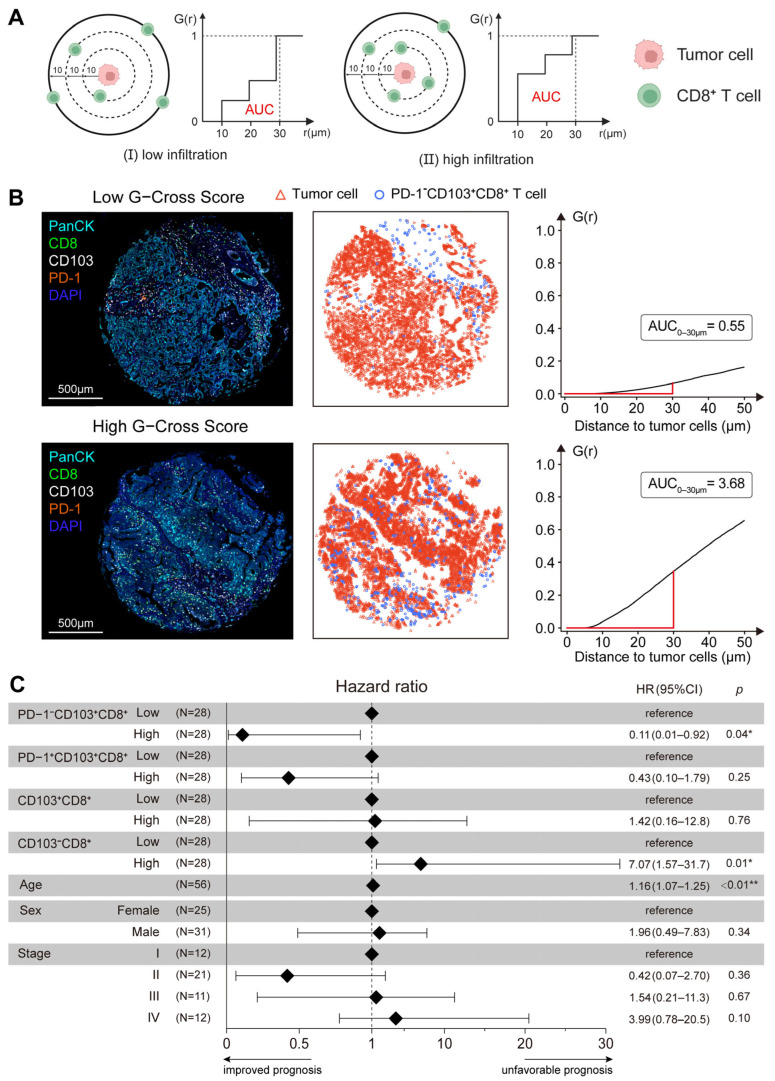
The spatial proximity of PD-1^−^ T_RM_ cells to tumor cells independently predicts favorable clinical outcomes. (**A**) Schematic representation of the G-cross function analysis used to quantify spatial proximity between tumor and CD8^+^ T cells within a radius *r* where the area under the curve (AUC) represents the proximity score of T cell infiltration. (**B**) Representative mIHC images with corresponding G-cross proximity scores quantifying the spatial relationship between tumor cells and PD-1^−^ T_RM_ cells. For spatial analysis algorithm requirements, quality control excluded samples with fewer than 1500 combined tumor cells and CD8^+^ T cells within tumor tissues (n = 5). (**C**) Forest plot of a multivariate Cox proportional hazards regression model including the clinical variables of sex, age, and stage alongside T cell spatial parameters demonstrating that high proximity of PD-1^−^ T_RM_ cells is a favorable prognostic factor (HR = 0.11, *p* = 0.04). (* *p* < 0.05, ** *p* < 0.01).

While spatial distance analysis quantifies the CD8^+^ T_RM_–tumor cell proximity to infer potential direct interactions, it inherently captures only pairwise relationships and cannot resolve distance-dependent spatial patterns. The G-cross function was further applied to characterize the radial organization and density gradients of CD8^+^ T_RM_ cells across multiple spatial scales comprehensively [22]. The probability of observing a CD8^+^ T cell within a defined radius (r) of a tumor cell was calculated by generating an area under the curve (AUC_0–30 μm_) score (Figure 5A). A dense accumulation of PD-1^−^ T_RM_ cells near tumor islets was exhibited by samples with high G-cross scores, whereas a sparse distribution distant from the tumor was shown by samples with low scores (Figure 5B). Multivariate Cox proportional hazards regression analysis was performed. Adjustments were made for sex, age, and tumor stage. The clinical independence of this spatial feature was validated. The analysis confirmed that a high proximity of the naïve T_RM_ subset served as a predictor of a favorable prognosis (hazard ratio [HR] = 0.11, 95% CI: 0.01–0.92, *p* = 0.04). Notably, there was no prognostic significance of the tissue-resident subset (CD103^+^CD8^+^) without PD-1 stratification (HR = 1.42, 95% CI: 0.16–12.8, *p* = 0.76), underscoring the critical necessity of distinguishing functional states within the T_RM_ compartment (Figure 5C). The G-cross method was validated for measuring the spatial proximity between subset T_RM_ cell types and tumor cells, which may serve as a potential prognostic factor in CCA. In the context of the tissue microenvironment, PD-1^−^ T_RM_ cells potentially possess features associated with better clinical outcomes.

### 3.6. Single-Cell Transcriptomics Reveal the Anti-Tumor Potential of PD-1^−^ T_RM_ Subsets

**Figure 6:**
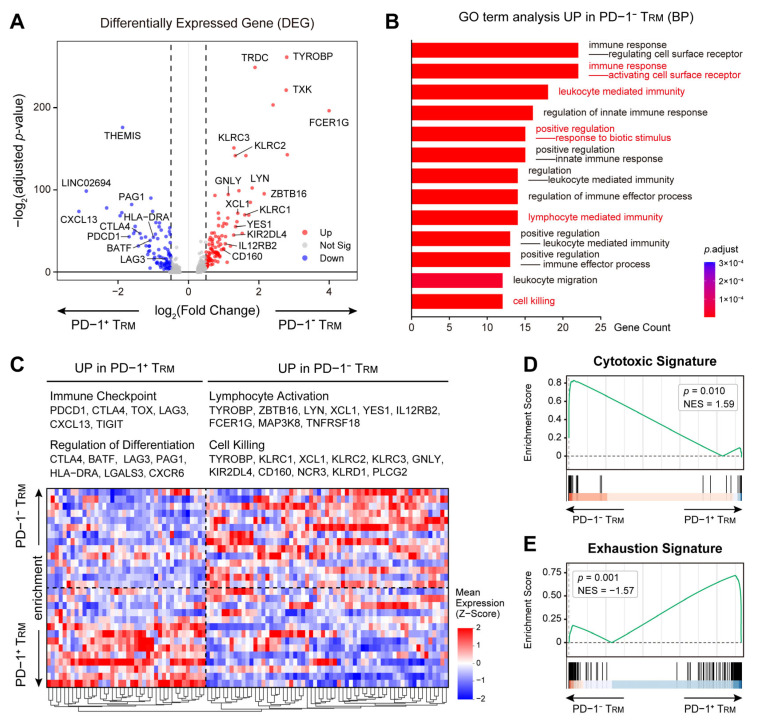
District gene expression profiles of PD-1^+^ T_RM_ cells and of PD-1^−^ T_RM_ cells. (**A**) Volcano plot showing differentially expressed genes (DEGs) between the PD-1^+^ and PD-1^−^ T_RM_ subsets, where key effector genes, including TYROBP and FCER1G, are upregulated in the PD-1^−^ T_RM_ subset, whereas checkpoint genes, such as PDCD1 and CXCL13, are upregulated in the PD-1^+^ T_RM_ subset. (**B**) Bar plot quantifying the top enriched Gene Ontology (GO) biological process terms in the PD-1^−^ T_RM_ subset, highlighting pathways associated with lymphocyte-mediated immunity, immune effector processes, and cell killing. (**C**) Heatmap displaying the scaled expression of key genes governing checkpoints, T cell differentiation, lymphocyte activation, and cell killing across the PD-1^+^ and PD-1^−^ T_RM_ subsets. (**D**) Gene Set Enrichment Analysis (GSEA) plot demonstrating significant enrichment of the cytotoxic signature in the PD-1^−^ T_RM_ subset (NES = 1.59, *p* = 0.010). (**E**) GSEA plot indicating enrichment of the exhaustion signature in the PD-1^+^ T_RM_ subset (NES = −1.57, *p* = 0.001).

The molecular mechanisms underlying the divergent prognostic impacts of T_RM_ subsets were elucidated through the analysis of the single-cell transcriptional profiles of PD-1^−^ versus PD-1^+^ T_RM_ cells. Differential expression gene (DEG) analysis uncovered a functional dichotomy between these two populations (Figure 6A). The PD-1^−^ T_RM_ subset was significantly enriched with genes associated with lymphocyte activation, such as TYROBP, ZBTB16, FCER1G, and genes related to cell killing, including KLRC1, KLRD1, PLCG2, consistent with the effector-competent phenotype (Figure 6A,C). The PD-1^+^ T_RM_ subset exhibited a robust upregulation of immune checkpoint molecules, such as PDCD1, CTLA4, LAG3, TOX, and transcription factors involved in T cell differentiation and exhaustion, such as TGFBR2, PRDM1, FOXP1, supporting a profile aligned with terminal exhaustion (Figure 6A,C; Table S3). Gene Ontology (GO) enrichment analysis revealed that the PD-1^−^ T_RM_ subset was functionally enriched in pathways related to the immune response, leukocyte-mediated immunity, and cell killing (Figure 6B; Table S4). Moreover, Gene Set Enrichment Analysis (GSEA) revealed a significant enrichment of the cytotoxic signature in the PD-1^−^ T_RM_ subset (NES = 1.59, *p* = 0.010, Figure 6D), whereas the exhaustion signature was enriched in the PD-1^+^ subset (NES = −1.57, *p* = 0.001, Figure 6E). This transcriptomic analysis identifies a transcriptional distinction between PD-1^−^ and PD-1^+^ T_RM_ subsets and provides a biological basis for their classification within the TME. These findings contribute to understanding the distinct roles of T_RM_ subsets with tumor immunity, which could potentially have significant implications for therapeutic targeting.

## 4. Discussion

**Figure 7:**
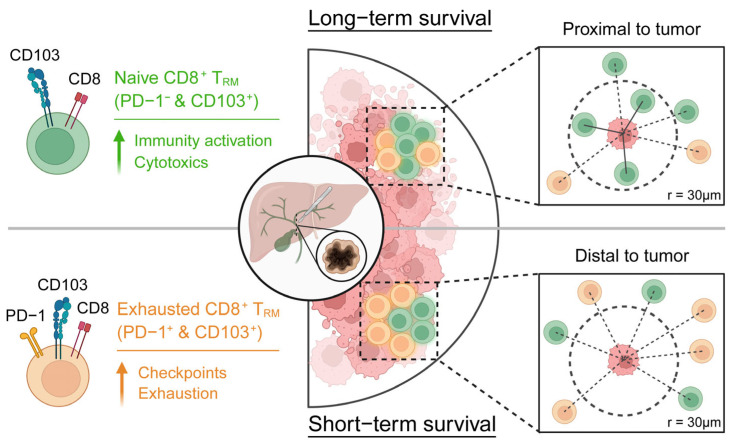
The immune landscape of the TME in CCA demonstrates distinct spatial distribution patterns of CD8^+^ T cell subsets between long-term and short-term survivors. Long-term survivors exhibit enrichment of PD-1^−^CD103^+^CD8^+^ T cells adjacent to tumor cells, where these cells actively mediate anti-tumor immunity.

This study employs mIHC and scRNA-seq to comprehensively characterize CD8^+^ T_RM_ cells in CCA, with a specific focus on PD-1 expression and their spatial distribution within the TME. Although CD103^+^CD8^+^ T_RM_ cells have been consistently associated with favorable outcomes across multiple solid tumors, their precise role in anti-tumor immunity—and particularly their contribution to therapeutic responses to PD-1 blockade—remains incompletely understood and is an emerging focus of research. In our cohort, a substantial proportion of PD-1^+^CD103^+^CD8^+^ T_RM_ cells are present within tumors; however, their presence is not associated with improved survival. Instead, we observe that exhausted T_RM_ cells (PD-1^+^) show reduced interactions with tumor cells, whereas naïve T_RM_ cells (PD-1^−^) exhibit increased interactions, which correlates with improved clinical outcomes. Importantly, the close proximity of PD-1^−^CD103^+^CD8^+^ T_RM_ cells to tumor cells serves as a predictor of a favorable prognosis. Single-cell analyses further support the anti-tumor potential of PD-1^−^CD103^+^CD8^+^ T_RM_ cells, suggesting a contribution to immunological surveillance and potential relevance to immunotherapy response (Figure 7).

This distinction between PD-1^−^ and PD-1^+^ T_RM_ subsets underscores a fundamental functional dichotomy within the tissue-resident compartment, where both subsets retain the core residency program, yet diverge in effector state and tumor engagement due to the influence of exhaustion-related molecules. Consistent with prior observations that T_RM_ populations in diverse solid tumors frequently express multiple inhibitory immune checkpoint molecules—reflecting progressive dysfunction under chronic antigen stimulation [23]. Nevertheless, our PD-1^+^ subset exhibits a transcriptional signature linked to exhaustion and dysfunction, most probably under the influence of chronic antigen pressure in the suppressive tumor microenvironment [4]. Conversely, the PD-1^−^ subset is characterized by a more polyfunctional, cytotoxic phenotype, which we suggest may be associated with ongoing anti-tumor immunity. These findings are conceptually aligned with evidence from other tumor types showing that tumor-localized CD103^+^CD8^+^ T_RM_ cells often co-express canonical residency markers (e.g., CD49a and CD69) and can exhibit high PD-1 expression, distinguishing them from CD103^−^CD8^+^ TILs in terms of differentiation and checkpoint engagement [24].

From an evolutionary perspective, the co-adaptation of tumors and the immune system has occurred. Tumor-specific T_RM_ cells can be generated after the effector response against the initial immunogenic cancer cells. During the equilibrium phase of tumor development, T_RM_ cells have been implicated in controlling tumor growth [25]. However, tumors can evade immune surveillance, including that from PD-1^−^CD103^+^CD8^+^ T_RM_ cells to PD-1^+^CD103^+^CD8^+^ T_RM_ cells, through mechanisms that may involve their transition toward a PD-1^+^ exhausted state and the creation of an immunosuppressive microenvironment. Notably, our spatial analysis indicated that the association with a functional phenotype occurs predominantly when these cells are in close physical proximity to tumor cells, which may suggest localized effector activity, such as cytolytic contact or cytokine secretion. This finding indicates that the spatial proximity metric is a potentially valuable prognostic measure even without standard clinicopathological variables, thereby underscoring a critical molecular-level pathological perspective that considers spatial biology in identifying future immune biomarkers. Instead of merely “having the right cells” for successful immune surveillance, it appears that a strategy involving “the right cells in the right place” may be relevant. This paradigm shift from purely immunological metrics to spatially defined molecular targets within the TME exhibits how specific molecules directly govern immune cell residency, adhesion dynamics, and functional activation or exhaustion states. Consequently, it is also essential for explaining the loss of prognostic significance for the total T_RM_ density, which could potentially offer prognostic benefits, when this beneficial subset is diluted or spatially separated from its target [26].

The relevance to developing immunotherapy is both significant and translational. First, the abundance and relative location of PD-1^−^ T_RM_ cells in relation to tumors could serve as a prognostic biomarker for an overall favorable outcome. Thus, it may help distinguish patients with a pre-existing spatially effective immune response [27]. Second, and more importantly, our data inform mechanistic hypotheses for immune checkpoint blockade. Although T_RM_ cells frequently express PD-1, it remains unclear whether PD-1^+^ versus PD-1^−^ subsets within the CD103^+^CD8^+^ compartment differentially shape responsiveness to anti-PD-1 therapy [24]. Notably, favorable prognosis in leukemia patients (without anti-PD-1 treatment) has been associated with a higher proportion of CD103^+^CD8^+^ T cells exhibiting relatively low expression of both PD-1 and TIGIT [28], consistent with our observation that PD-1^−^ T_RM_ cells in close proximity to tumor cells predict better outcomes in CCA. Moreover, co-expression of immunosuppressive receptors such as PD-1 and TIGIT has been linked to impaired effector function, while dual inhibition of PD-1 and TIGIT can significantly enhance CD8^+^ T cell cytotoxicity [29]. Together, these findings suggest that PD-1 may serve as a reference marker for a therapeutically relevant T_RM_ subset, yet functionally constrained population that could be re-invigorated by checkpoint blockade. Therefore, by blocking the activation of the PD-1 signaling, the therapy might partially reverse the exhausted phenotype of PD-1^+^ T_RM_ cells [30]. This modulation may be accompanied by changes in their spatial behavior, potentially driving a phenotypic shift toward a PD-1^−^ T_RM_-like state and promoting their redistribution into closer proximity to tumor cells. This spatial reprogramming may be an essential element in successful treatment.

Future studies ought to conduct in-depth investigations into the signals that regulate the spatial positioning of T_RM_ subsets within tumors [31,32]. Ultimately, it is yet to be ascertained whether therapeutic strategies, when combined with checkpoint blockade therapy, which involve using chemokines or other modulators to actively recruit T_RM_ cells into the TME or to stably implant functionally competent T_RM_ cells in the vicinity of tumor cells, can prove effective [33,34]. Overall, our study presents a comprehensive spatial-functional profiling of T_RM_ cells, providing a crucial framework for comprehending anti-tumor immunity and informing the development of next-generation immunotherapies for CCA, as well as potentially other solid malignancies.

## 5. Conclusions

In summary, our study identified distinct subsets of CD8^+^ T cells in CCA with prognostic significance based on their activation state and spatial distribution. Naïve T_RM_ cells (PD-1^−^, CD103^+^, CD8^+^) interact more with tumor cells and correlate with favorable survival, whereas exhausted T_RM_ cells (PD-1^+^, CD103^+^, CD8^+^) show fewer interactions. The proximity of PD-1^−^CD103^+^CD8^+^ T_RM_ cells to tumor cells is linked to improved clinical outcomes. Single-cell analysis revealed that these cells expressed genes related to lymphocyte activation and cytotoxicity. These findings suggest that PD-1-targeted immunotherapies may influence anti-tumor immunity through altering the spatial localization of exhausted T_RM_ cells.

## Data Availability

The raw data supporting the conclusions of this article will be made available by the authors on request.

## Supplementary Materials

The following supporting information can be downloaded at: https://www.mdpi.com/article/10.3390/cancers18040680/s1, Figure S1: Comparison of T cell subpopulations in tumor and adjacent normal tissues; Figure S2: High infiltration of exhausted PD-1^+^CD8^+^ T cells in the whole TMA section indicates poor prognosis of cholangiocarcinoma; Figure S3: Represent single-channel images of PD-1^−^ T_RM_ and PD-1^+^ T_RM_ cells from CCA patients with short-term and long-term survival; Table S1: Characteristics of patients; Table S2: Panel information; Table S3: Differentially Expressed Gene between T_RM_ subsets; Table S4: GO enrichment analysis of PD-1^−^ T_RM_ (BP) subsets; Table S5: Original and FDR-adjusted *p*-values for statistical tests.

## Abbreviations

The following abbreviations are used in this manuscript:

CCA	Cholangiocarcinoma
mIHC	Multiplex immunohistochemistry
scRNA-seq	Single-cell RNA sequencing
T_RM_	Tissue-resident memory T
TME	Tumor microenvironment
HR	Hazard ratio
