# Supplementary figures and images for: The Proximity of PD-1−CD103+ Tissue-Resident CD8+ T Cells to Tumor Cells Is Correlated with Improved Clinical Outcomes in Patients with Cholangiocarcinoma
Source: Cancers (Basel). 2026 Feb 19;18(4):680. doi: 10.3390/cancers18040680 (PMC12939198; doi:10.3390/cancers18040680)

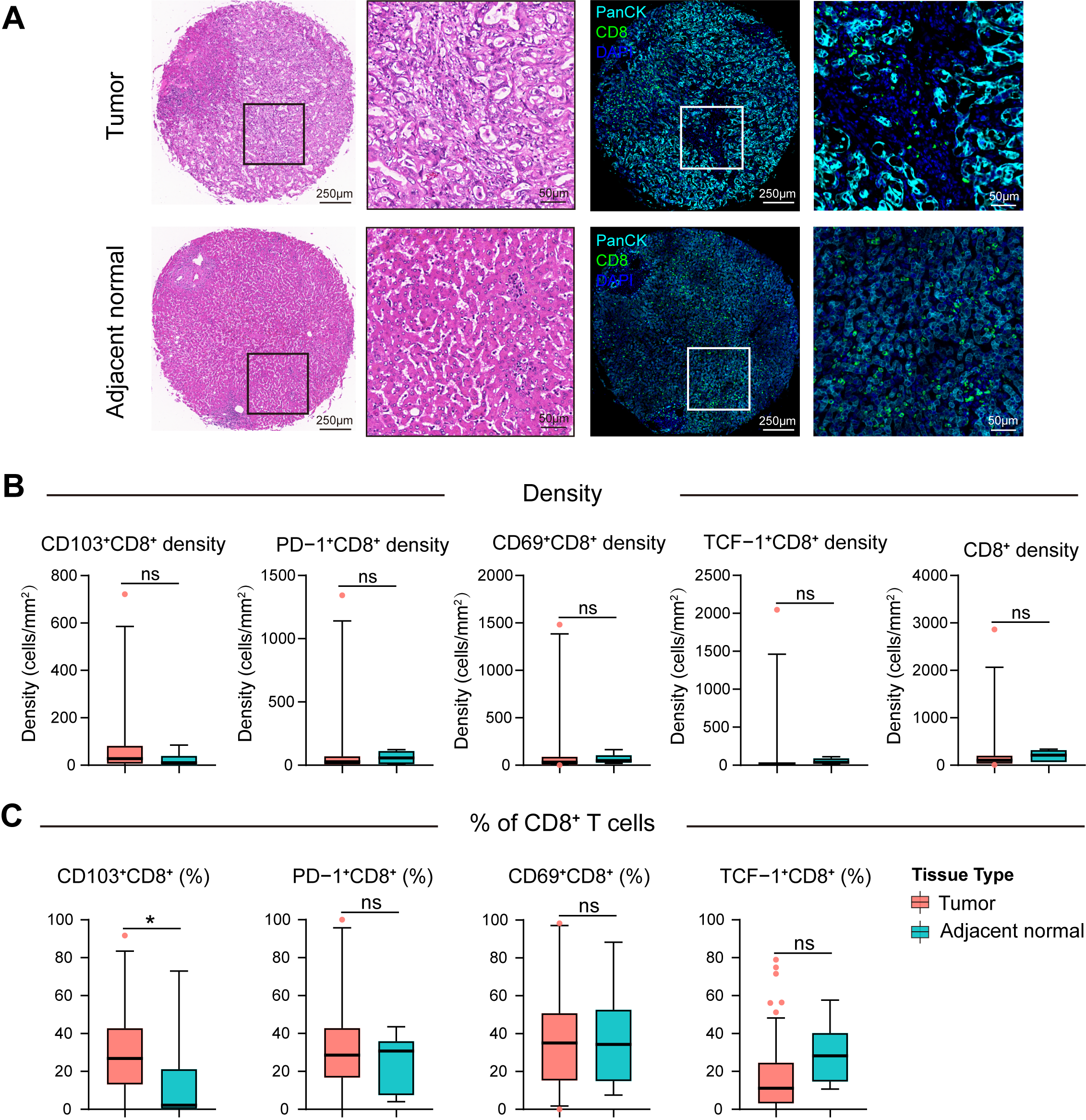

Supplement: Supplementary file 1 [file cancers-18-00680-s001.zip › Figure S1.png]

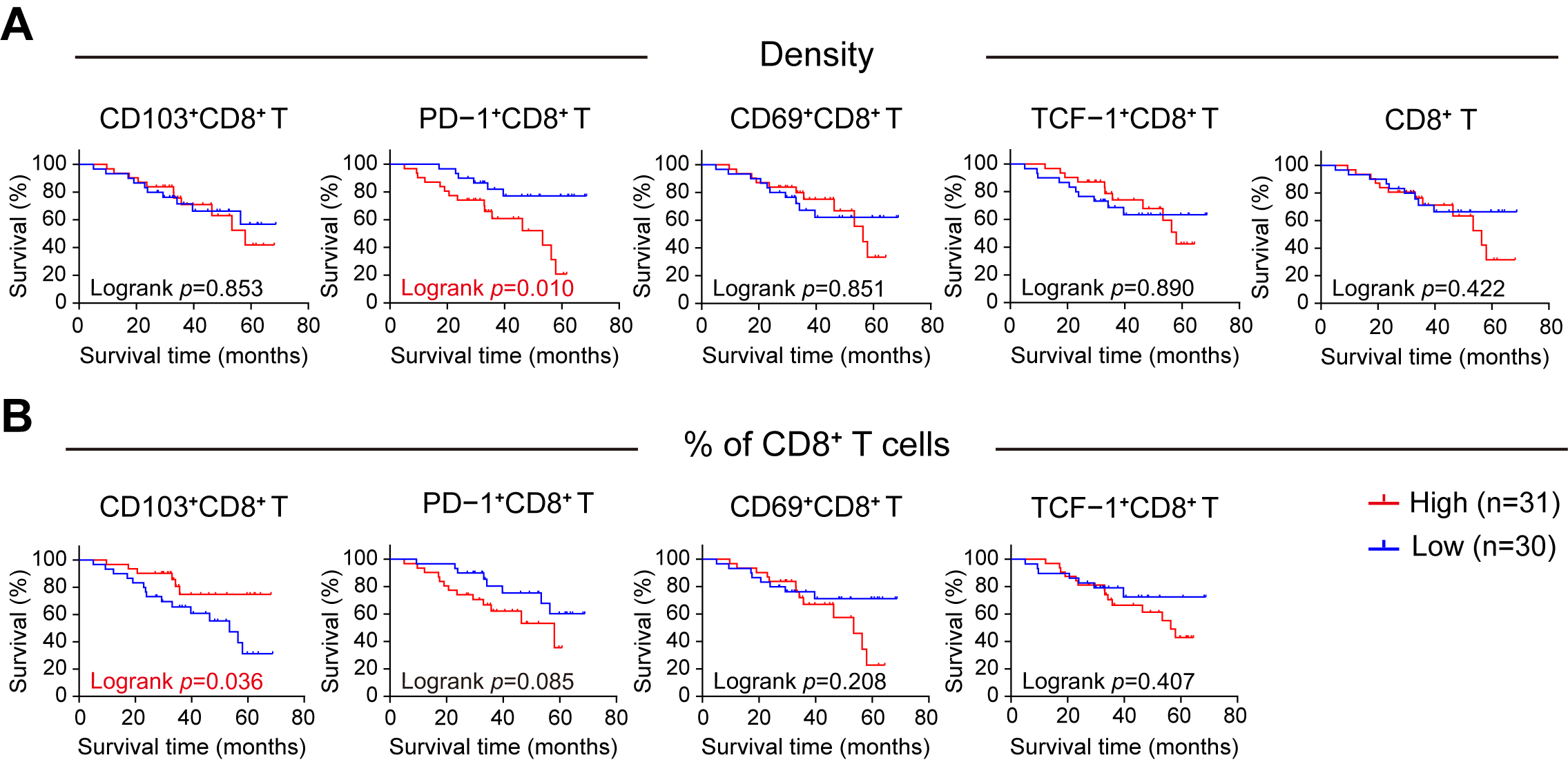

Supplement: Supplementary file 1 [file cancers-18-00680-s001.zip › Figure S2.png]

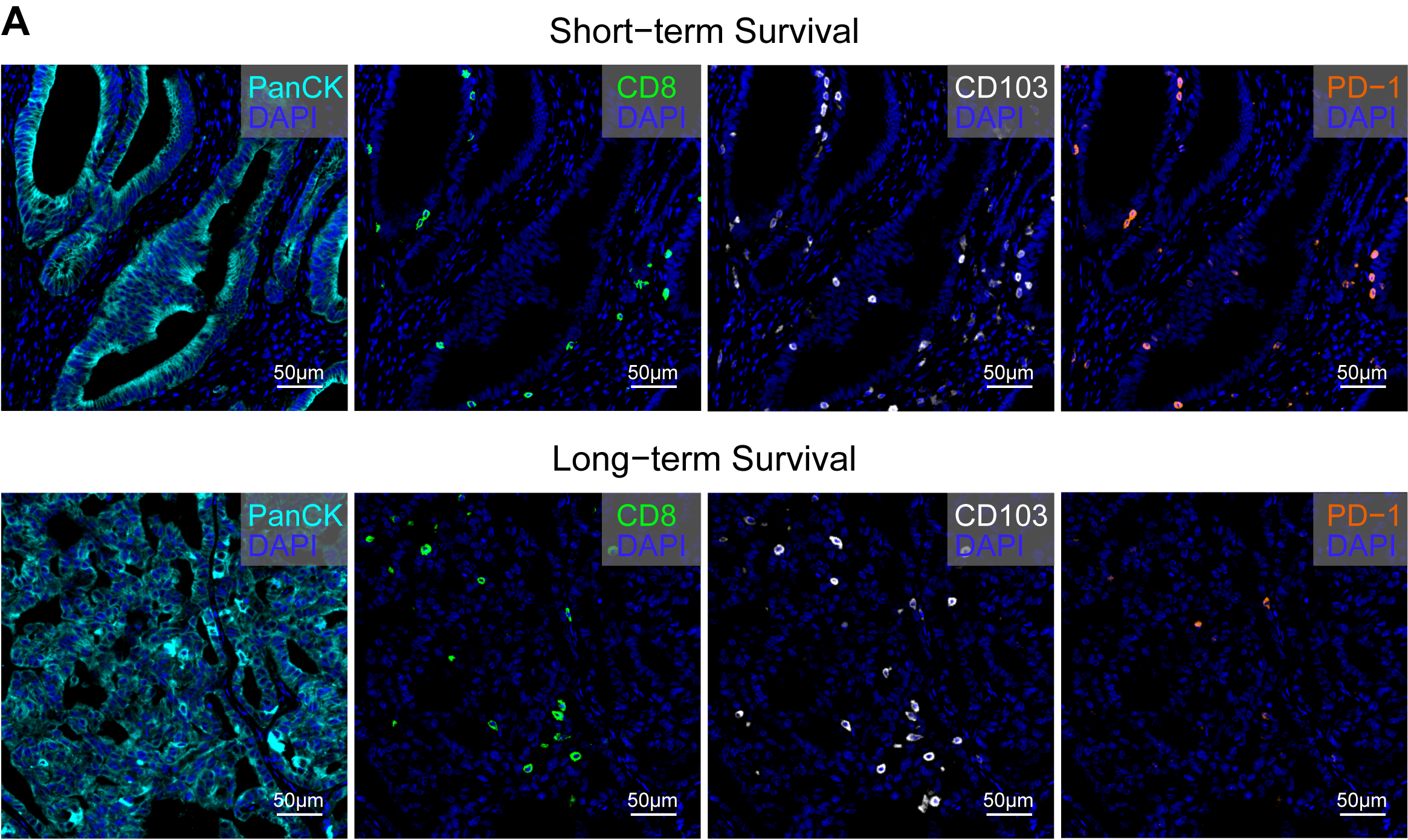

Supplement: Supplementary file 1 [file cancers-18-00680-s001.zip › Figure S3.png]
